# Universal health coverage in Lebanon: agenda setting using Kingdon's model and a proposed legal framework for revenue allocation

**DOI:** 10.3389/frhs.2025.1627319

**Published:** 2025-08-15

**Authors:** Joseph Mouawad, Maria Rita Lteif, Wadih Mina, Rita Karam

**Affiliations:** ^1^ Melbourne School of Population and Global Health, University of Melbourne, Melbourne, VIC, Australia; ^2^ Faculty of Health Sciences, American University of Beirut, Beirut, Lebanon; ^3^ Independent Researcher, Beirut, Lebanon; ^4^ Faculty of Sciences and Medical Sciences, Lebanese University, Hadath, Lebanon; ^5^ Quality Assurance of Pharmaceutical Products Department, Lebanese Ministry of Public Health, Beirut, Lebanon

**Keywords:** agenda setting, Kingdon model, Bardach & Patashnik's framework, policy analysis, universal health coverage, excise taxes, earmarked taxes, Lebanon

## Abstract

**Background:**

Health systems globally aim for Universal Health Coverage (UHC), however progress toward UHC remains challenging in many countries. Despite previous unsuccessful parliamentary attempts to secure national commitment to a UHC plan in Lebanon, a renewed and revised effort is currently being made by a dedicated parliamentary committee. This study has two main objectives: to examine the emergence of the UHC bill as a priority on the Lebanese policy agenda using John Kingdon's Framework; and (2) to conduct a policy analysis using Bardach and Patashnik's eight-step framework to identify the most suitable funding option for the proposed UHC program.

**Methods:**

Two policy analysis frameworks were applied: John Kingdon's model, commonly used to study agenda setting, and Bardach and Patashnik's eight-step framework, designed to evaluate policy options and recommend evidence-based solutions. A qualitative document analysis was conducted using the READ approach drawing on secondary literature sources. Data triangulation was also used to ensure consensus and accuracy.

**Results:**

The document analysis identified the emergence of a window of opportunity for the UHC bill as the three streams aligned: mounting public pressure, the formation of a parliamentary committee, and evident political receptiveness through the involvement of multiple parties. Bardach & Patashnik's policy analysis framework identified earmarked excise taxes as the most suitable policy alternative for financing UHC in Lebanon.

**Conclusion:**

This study explains the emergence of an updated UHC bill on the Lebanese policy agenda while conducting a policy analysis of the earmarked excise taxed to fund UHC. To overcome anticipated challenges a legislative framework must be established to ensure transparency in funding and government accountability.

## 1 Introduction

### 1.1 UHC and country context

A country's ability to provide equitable quality health services to its population without facing financial hardship, also known as UHC, is recognized as a core component of the Sustainable Development Goal (SDGs) (1). Successful UHC programs have also been the focus of several electoral campaigns and political promises, aiming to provide patients quality care where and when they need it without financial hardships (2–5). Unfortunately, the world is off track in making significant progress towards UHC by 2030, with developing countries lagging the most behind (1).

Lebanon, once renowned for having one of the most advanced healthcare systems in the Middle East, has been struck by a series of multifaceted crises since October 2019, leading to its downgrade from an upper-middle-income to a Lower-Middle-Income Country (LMIC) according to World Bank classifications (6–8). The country's economic collapse, severe depreciation of the Lebanese Lira, and depletion of the central bank's foreign currency reserves, have drastically strained the government's fiscal capacity. This has led to cuts in subsidies on essential goods and healthcare services, and an increasing inability to cover patients under the already fragmented healthcare system (9). These structural weaknesses were further intensified by the COVID-19 pandemic, which deepened societal vulnerabilities, heightened food insecurity, and placed additional strain on the privatized and expensive healthcare sector (10). The devastating Beirut Port explosion on 4 August 2020 compounded these challenges, destroying more than half of Beirut's health infrastructure and further limiting service availability (11).

As of 2023, significant barriers to affordable healthcare remain, with high out-of-pocket expenditures and multidimensional poverty affecting over 80% of the population, including dimensions related to health, employment, and availability of public utilities (12). The country is now experiencing reduced access to essential healthcare services, with less than half of the population receiving any form of healthcare coverage (13). Despite repeated electoral promises in Lebanon between 2000 and 2022, a UHC law has not yet been passed by the Lebanese Parliament, mainly due to poor governance, inadequate accountability mechanisms, and financial challenges (7, 13).

In May 2023, and for the first time, Lebanese Parliamentarians undertook a significant initiative by proposing a UHC bill to provide comprehensive and affordable care to the Lebanese population (14, 15). The articles under this UHC bill include providing primary health care services to all Lebanese citizens, including basic preventive and curative health services under the Ministry of Public Health's (MoPH) Primary Healthcare Network. A comprehensive benefit package that includes hospitalization, dialysis, and drugs for cancer and rare diseases will be offered to Lebanese who do not benefit from any official health coverage, whether in their personal capacity or as right seekers (14).

Financing this UHC program will come from various sources, including fees on wealth, cash transfers made by non-bank institutions, and excise taxes on harmful products such as tobacco and alcohol. These excise taxes are planned to range from 10% to 100%, with higher rates applied to imported goods, including 50% on imported tobacco, spirits, and cosmetics, and 100% on cigars. Locally produced items will be taxed at lower rates, with 25% on tobacco and spirits, and 10% to 50% on other products like energy drinks and cigars. Revenue from these taxes will be earmarked specifically for healthcare initiatives. Beneficiaries will also contribute to the system, though certain categories such as holders of a disability card issued by the Ministry of Social Affairs, will be exempt (14). To ensure proper governance a special committee will be established to manage the UHC body and coordinate with relevant stakeholders and organizations (14).

### 1.2 John Kingdon's multiple streams framework

Developed in the 1980s, the John Kingdon's Multiple Streams Framework (MSF) has become one of the most influential agenda-setting theories in policy analysis, used to retrospectively analyze why ideas receive attention over others within the policy environment (16, 17). Originally applied to U.S. federal policy-making, the MSF has since been adapted and applied across diverse national and sectoral contexts, including health policy, due to its flexibility in analyzing complex non-linear processes (18).

The MSF suggests that agenda setting occurs after the convergence of three streams: (1) **the problem stream**, which consists of a well-defined issue recognized by several actors, (2) **the policy stream**, which involves potential solutions to the policy problem provided by policy entrepreneurs, academicians, researchers, interest groups, etc., and (3) **the politics stream**, which considers the national environment as monitored by the government, media, and pressure groups (19–21). When conditions become favorable for all three streams to interact and align, a window of opportunity opens, increasing the chance for a policy to gain a place on the policy agenda (22). This requires policy entrepreneurs to match solutions to the problems, facilitate the process, and create opportunities for policy progress before it is lost (23, 24).

In the context of public policy, the agenda setting metaphor refers to the list of issues or problems that government officials and closely associated actors are giving serious and active attention to at a given point in time (17). The recent prioritization of a UHC bill on the Lebanese parliamentary agenda after a decade of promises can thus be explained using the MSF (16, 17).

### 1.3 Bardach and Patashnik's policy analysis framework

Achieving UHC as a national goal requires a robust legal framework, and excise taxes applied to products like tobacco, alcohol, and Sugar-Sweetened Beverages (SSBs) have been considered a revenue source to fund many healthcare systems globally (25). However, implementing them in Lebanon raises several legal questions, which require a proper policy analysis.

To address such complex policy issues, Bardach and Patashnik proposed a widely used, practical, and step-by-step policy analysis framework that is both accessible and effective, even without sophisticated analytical methods (26). The framework consists of eight steps: (1) **define the problem** — clearly identify and frame the policy issue; (2) **assemble evidence** — collect relevant data to understand the issue and inform options; (3) **construct alternatives** — develop a range of feasible policy solutions; (4) **select criteria** — establish indicators for evaluating alternatives; (5) **project outcomes** — estimate the likely effects of each option; (6) **confront trade-offs** — weigh the benefits and drawbacks of competing options; (7) **decide** — recommend the most appropriate course of action; and (8) **share results** — effectively communicate findings and recommendations to stakeholders (27).

Bardach and Patashnik's policy analysis framework has been widely applied in policy research, public health, and other diverse contexts as a comprehensive and practical, evidence-based tool for analyzing policy issues (28). It is particularly suitable for examining the proposed funding mechanisms for Lebanon's UHC bill, as it enables a thorough assessment of policy options, trade-offs, and implications within the country's unique socio-political and economic context.

Accordingly, this study applies steps 1–7 to conduct the analysis, with the findings and discussion presented here corresponding to step 8: communicating the results to inform policymakers and stakeholders.

This study has two main objectives. First, it examines the emergence of a UHC bill as a priority on the Lebanese policy agenda using the MSF. Second, it conducts a policy analysis to identify the most suitable funding option for the proposed UHC program, applying Bardach and Patashnik's eight-step framework. Accordingly, this paper is structured into two main sections, each addressing one of these objectives.

## 2 Methods

### 2.1 Study design

A qualitative policy analysis design utilizing a document analysis approach was used. It combines two established frameworks: John Kingdon's MSF to understand how the UHC bill was prioritized on the Lebanese policy agenda, and Bardach & Patashnik's policy analysis framework to strategically identify the most suitable funding option for the proposed bill from a legal perspective.

### 2.2 Theoretical frameworks

As agenda setting is among the first steps in the policy development process, the MSF framework was used to understand how this UHC bill was put forth on the Lebanese policy agenda (29). This framework has contributed to identifying how certain issues or policies make it onto the decision-making agenda in complex political environments (16) and has become widely used in LMICs (30, 31). Examples include the passage of the tobacco control law in Lebanon (32), prioritizing mental health in Africa (33), and considering gender-based violence as a health issue in Nepal (29).

Bardach & Patashnik (2023) framework for policy analysis was further adopted, focusing on the content analysis of the UHC bill, examining the provisions related to excise taxes, and their role in financing UHC. The steps of Bardach and Patashnik's framework align with the study objectives and take into account data availability. The analysis concludes with a summary of findings, actionable recommendations, and a concise conclusion that connects the policy implications.

### 2.3 Document analysis and data sources

This study relied on high-quality secondary data from the literature which also include content from the official UHC bill issued by the Lebanese Parliament in May 2023. At the time of this study, the UHC bill consisted of 7 chapters and 23 articles which were thoroughly examined to identify provisions related to excise taxes, including their rates and the proposed mechanisms for allocation. This is a working document, and further versions may emerge as the policy process evolves, reflecting ongoing negotiations and adjustments that could affect the bill's final content and implementation.

Secondary sources of data were retrieved online from scientific databases such as PubMed and Google Scholar, along with grey literature reports, books from government agency websites, and university archives. The Keywords used were: “universal health coverage”, “economic crisis”, “health taxes”, “excise taxes”, “Lebanon”, “earmarking”, “health financing”, “policies”, “public health”, “health policy”, “legal framework”. Boolean operators were used to link concepts and combine key terms in the search engines.

To ensure procedural rigor, the four-step READ approach was applied for document analysis: Ready materials, Extract data, Analyze data, and Distil findings (34). In step 1, after the search analysis yielded several results, specific inclusion criteria were applied. Studies needed to address events following the 2019 crisis in Lebanon, to focus on political, health policy, or economic perspectives, to align with the two analytical frameworks used, and to primarily examine Lebanon or other LMICs with similar contexts. Documents unrelated to UHC, health policies, and excise taxes were excluded. Articles that solely discussed general taxation without linking it to health outcomes, healthcare financing models without reference to excise taxes, or excise taxes in non-health-related contexts (e.g., environmental or trade policies) were also excluded. Additionally, opinion pieces and studies lacking empirical data or policy relevance were excluded. The selection was further refined by removing duplicate and non-essential articles. The final dataset included scientific and peer-reviewed publications, books, official documents (such as policy directives, statements, and declarations), policy briefs, policy dialogues, newspaper and magazine articles, podcasts, meeting reports, grey literature, conference proceedings, workshop summaries, legislative bills and drafts, technical reports, case studies, and evaluation reports.

In step 2, data were systematically extracted using a standardized Excel sheet that included key study characteristics such as country, database, authors, study title, source, population data, document type, and relevant keywords. Extracted data were categorized under Kingdon's Multiple Streams Framework when applicable and aligned with Bardach & Patashnik's policy analysis framework.

Step 3, elaborated below, involved an analytical and iterative approach to evaluating and synthesizing the retrieved data. Inclusion and exclusion decisions were continuously refined based on relevance, emerging insights, and the strength of evidence.

In step 4, an iterative review and integration of findings led to data saturation, enhancing the comprehensiveness and robustness of the analysis presented in this article.

### 2.4 Data analysis

As this was an iterative qualitative process, the documents collected were analyzed thematically and categorized under key themes related to excise taxes, such as their impact on public health, revenue generation, and successful implementation strategies in other countries. Chronological patterns of events were also analyzed, considering the complexity of the Lebanese context, to clearly identify the three streams of the MSF and their alignment. A comparative analysis was also conducted, examining Lebanon's proposed UHC policies against successful models from other countries. This helped identify potential lessons and best practices to feed step 2 of the policy analysis framework “assembling evidence”. Finally, data triangulation was achieved by incorporating and validating information from multiple sources (34).

## 3 Results

### 3.1 John Kingdon's multiple streams framework

The results of the document analysis were mapped under the three streams of the Kingdon Model:

#### 3.1.1 The problem stream

The multifaceted crises Lebanon has been facing since 2019 have pushed the health system to the brink of collapse, particularly due to the economic crisis, which has severely damaged the economy and brought disastrous consequences to the Lebanese population (13, 35). This economic crisis has been described by the World Bank as one of the 10 worst global crises since the mid-19th century, leading to a 40% decline in the country's GDP in 2020, an average inflation rate of 172% in 2023, and Lebanon's reclassification as a lower middle income country (8, 36, 37). Basic goods, including food, fuel, and medicine, were originally subsidized by the Central Bank as a form of support for socio-economically vulnerable populations (38). However, with the strain on Lebanon's economy, the Central bank could no longer continue with these subsidizes, increasing Out Of Pocket (OOP) expenditure to more than 80%, way above the WHO's recommended limit for catastrophic health spending (39, 40). The MoPH has since stated that the government reduced its total spending on the health sector by 40% between 2018 and 2022 (41). As a result, a decline in healthcare access was witnessed, with the percentage of households struggling to access healthcare rising from 25% to 36% within approximately four months in 2020, hospitalizations decreasing by 30% in 2021, and average monthly hospitalization days dropping by 25% (42). The COVID-19 pandemic was also identified as a national priority by the MOPH necessitating the need to respond quickly to the spread of transmission and reducing the number of deaths (43). Amid an already strained healthcare sector, the government collaborated with international Non-Governmental Organizations (iNGOs), municipalities, and the private sector to implement lockdowns, travel restrictions, and a phased national vaccination plan aimed at achieving 70% coverage by the end of 2022. However, structural challenges, including weak epidemiological surveillance and limited healthcare resources caused some delays in implementation (43).

Policymakers, academicians, knowledge translation centers, and journalists have also published statements around the deterioration of the healthcare system in Lebanon. They have identified unimaginable levels of suffering for the population in trying to access their medications and a combination of social, economic, financial, political and healthcare challenges (41, 43). Local and international NGOs have also observed increased vulnerability among their patients who are unable to afford and access care, forced to skip chronic medications and routine medical checkups (44, 45). These iNGOs have increased financial aid and support during this time, particularly within the Primary Healthcare Network and Governmental hospitals through providing fuel, medical supplies, and financial coverage (46).

Government officials, policy makers, and international organizations are not the only stakeholders who have identified this problem, Lebanese citizens and more specifically patients, have expressed their struggles. Cancer patients reported skipping their treatment due to shortages, patients with autoimmune diseases resorted to buying their medications from abroad, and patients on chronic medications are facing availability challenges (41). Patients protested in the country as a call for action amidst their struggles, supported by NGOs, influencers, and syndicate presidents (47). Healthcare professionals (HCPs) including doctors and pharmacists have also spoken up about medical shortages and expenses, with the president of the Lebanese Pharmaceutical Importers Association stating that import companies' debts have reached 400 million dollars (9).

All these actors have recognized the inequitable and deteriorating healthcare system as a major problem in the country, pushing patients into suffering, and straining healthcare facilities further, even years after the 2019 crisis.

#### 3.1.2 The policy stream

The policy stream in Lebanon involved a collaborative effort to identify and implement viable solutions to enhance healthcare access and achieve UHC. These solutions are tailored to align with Lebanon's sociocultural context, budgetary constraints, and political feasibility, increasing their chances of acceptance and integration into the policy agenda. In January 2023, the MoPH, in cooperation with the WHO, launched the National Health Strategy - Vision 2030, prioritizing UHC as a key approach to address rising unmet health needs and strengthening the healthcare system (42). This strategy, developed through a collaborative process involving high-level experts and parliamentarians, aims to address rising unmet health needs and to strengthen the healthcare system. A dedicated committee at the level of parliament, which includes representatives from key ministries, is meeting regularly to deliberate on the proposed UHC bill and address critical issues related to its implementation and funding (13). In line with this objective and to respond to the ongoing health crisis, the World Bank introduced the Health Transformation Project later in 2023, positioning UHC as a strategic solution to prevent the collapse of the health system (35). Furthering these efforts, the MoPH, in partnership with the World Bank, organized a workshop in February 2024 with various stakeholders to promote and advocate for UHC, aiming to create a unified vision for comprehensive health coverage (13, 35, 42).

At the forefront of these efforts, the Knowledge to Policy (K2P) center at the American University of Beirut, recognized by the WHO for its evidence-based approach, has been pivotal in shaping solutions for UHC (48). It organized a policy dialogue focused on financing UHC, which included 21 stakeholders from diverse backgrounds such as government, NGOs, researchers, and the pharmaceutical sector. This dialogue led to consensus on implementing excise taxes and redirecting revenues to fund UHC, offering clear, actionable recommendations such as forming a core group of experts to draft a UHC law (49). Complementing these initiatives, media campaigns have advocated for UHC as a solution to Lebanon's healthcare crisis (50). For instance, K2P launched “Policy Corner” a series of episodes on key health policy topics, shared on social media platforms to raise awareness and increase public understanding of UHC (48).

#### 3.1.3 The politics stream

The movement for UHC has gained global momentum, with UHC becoming an important part of the SDGs, particularly SDG 3.8. In September 2023, the United Nations adopted a high-level political declaration to reaffirm and renew its commitment towards UHC after the COVID-19 pandemic (51).

Despite successive acknowledgments by Lebanese governments on the importance of UHC, little progress towards commitment was previously seen. In recent years, however, UHC has gained prominence in electoral platforms (13). The most recent UHC bill, initially introduced by a Member of Parliament (MP) from a major political group, was later amended by MPs from another political party in October 2018. By June 2022, the Chairman of the Parliamentary Health and Social Affairs Committee (PHAS), who belonged to a different political party, took a leading role in promoting the bill within Parliament. His efforts resulted in the formation of subcommittees representing various political groups to review the proposed legislation (14). This development reflects increasing political will and suggests new efforts toward concrete healthcare reforms. Additionally, policymakers, including the Minister of Health, have strongly committed to advancing UHC (42).

The severe financial crisis in Lebanon has reshaped the political landscape, weakening traditional patronage networks and forcing political elites to seek alternative ways to maintain support from their followers (52). The deteriorating healthcare system has affected all social groups, including those aligned with different political factions, creating pressure on politicians to endorse reforms that would provide tangible benefits to their voters (52). Given Lebanon's reliance on external financial and technical support (53), endorsing UHC aligns with the broader strategic interests of political leaders who seek to secure international aid while responding to domestic demands. UHC has thus emerged as a rare point of convergence among feuding political groups and as a politically viable solution in addressing urgent healthcare needs across different constituencies.

**Figure 1:**
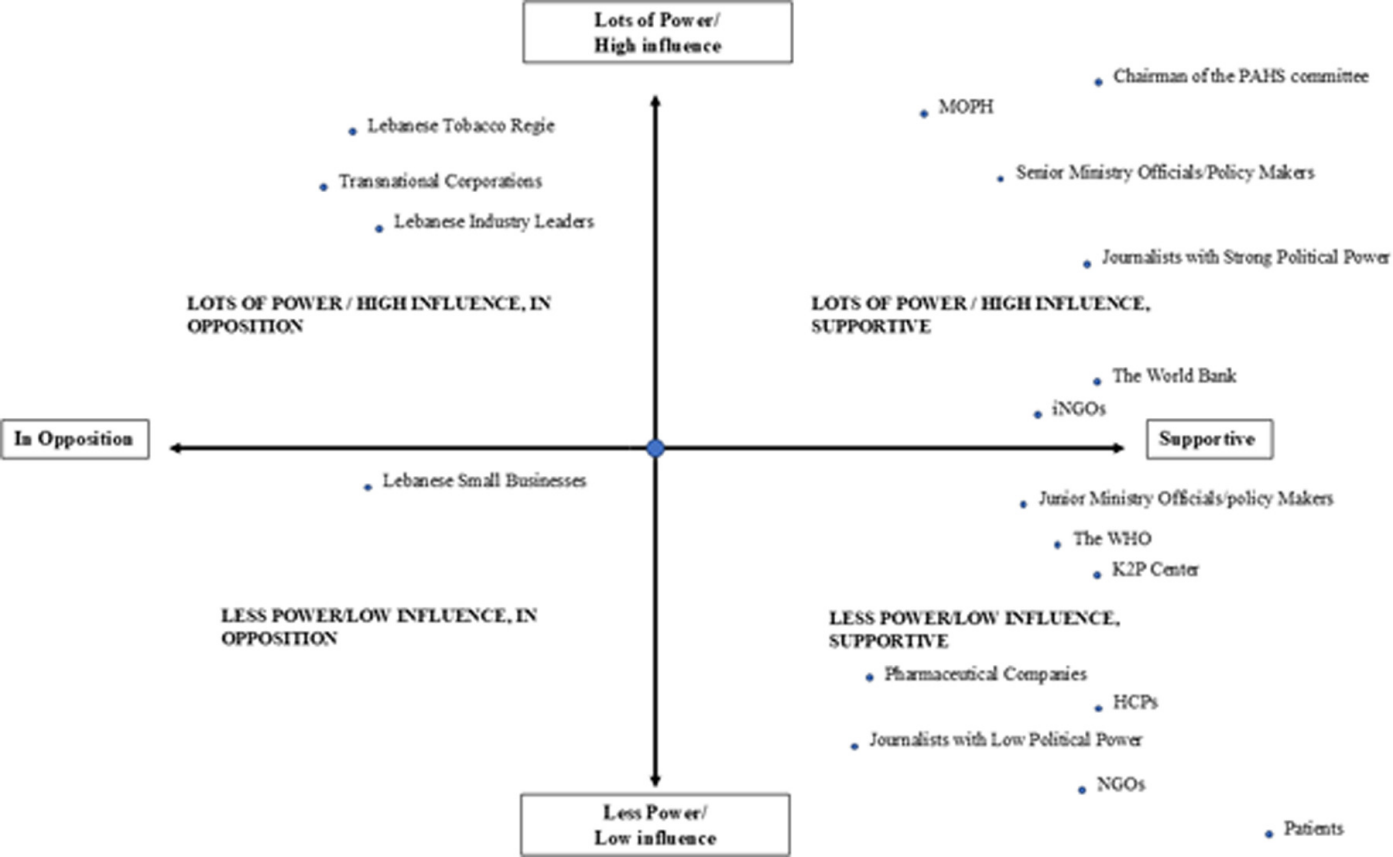
Power analysis matrix of the different stakeholders identified by the MSF. **Lots of Power/High Influence, In Opposition**: Features the Lebanese Tobacco Regie, affected Transnational Corporations (TNCs), and Lebanese Industry Leaders, who hold substantial influence but would oppose this UHC bill due to increased tax rates and potential revenue losses. **Lots of Power/High Influence, Supportive**: Includes senior policymakers like the Chairman of the PHAS Committee, Ministry of Public Health (MoPH), Ministry Officials, The World Bank, and iNGOs reflecting their strong support and ability to drive UHC initiatives. **Less Power/Low Influence, In Opposition**: Represents Lebanese Small Businesses, which have minimal influence and oppose the UHC policy, likely due to concerns over increased regulations and costs, despite facing a comparatively smaller tax rate. **Less Power/Low Influence, Supportive**: Comprises Pharmaceutical Companies, Junior ministry officials/policy makers, Healthcare Professionals (HCPs), journalists with low political power, the WHO, k2P, NGOs, and Patients, indicating limited influence but support for UHC.

After the current alignment of the politics, policy, and problem streams, along with concentrated efforts by stakeholders, a window of opportunity opened and the UHC bill is in the forefront of the Lebanese policy agenda, just a few steps away before it becomes a law. The main Policy entrepreneurs (Figure 1) behind the UHC bill in Lebanon are ministry officials, parliamentarians, the MoPH, the PHAS, the K2P center, the WHO, the World Bank, journalists, pharmaceutical companies, and active HCPs within the country. Different forms of power exist among these stakeholders. For instance, entities like K2P excel in knowledge production and advocacy, while journalists derive their political influence from their affiliations, audience reach, or the political backing they receive. The same applies to policymakers and parliamentarians, given Lebanon's sectarianism and varied levels of power. NGOs' influence also depends on whether they are international NGOs holding foreign expertise and funds, or local NGOs who have limited resources to work with. Which is why these stakeholders were subdivided into categories.

Nonetheless, all these stakeholders were involved in defining the problem, participated in proposing solutions, and actively pushed for UHC as a necessary reform to address Lebanon's healthcare crisis. Their efforts included raising awareness through media campaigns, organizing multi-stakeholder workshops, and collaborating on policy discussions to ensure that UHC remained a top priority. These policy entrepreneurs are key entities that have either consistently advocated for evidence-based policymaking, leveraged political connections and negotiation skills, or demonstrated their commitment to UHC reform, thereby creating a window of opportunity for policy change.

### 3.2 Bardach & Patashnik's policy analysis framework

#### 3.2.1 Step 1: defining the problem

As described in the problem stream above, Lebanon's healthcare system has been severely impacted by the multifaceted crises since 2019. The Central Bank's withdrawal of subsidies, coupled with government budget cuts, has led to a loss of financial protection, leaving many patients unable to afford essential healthcare services, further worsening the situation for both patients and healthcare facilities.

Despite the Lebanese Parliamentary Health Committee's introduction of a revised version of the UHC bill, it still contains major deficiencies and gaps. The evidence used to determine the recommended tax rates on tobacco, alcohol, and SSBs remain unclear. The strategic objective SO.2.1.3 within the Lebanese National Health Strategy focuses on implementing a real-term increase in health financing through taxes, with adequate allocation to primary healthcare and a reduction in out-of-pocket expenses (42). However, the strategy does not include a clear funding mechanism detailing how the costs of UHC will be covered and sustained over time. This strategy may encounter challenges in Lebanon due to the existing legal framework that prohibits the earmarking of funds (49).

Furthermore, the problem is worsened by the inadequately proposed low levels of taxation for tobacco and alcohol products (Article 7 of the Bill) (14). For example, the excise tax proposed on cigarettes is 25% on all types of locally produced tobacco and around 50% on all types of imported tobacco. This is lower than the World Health Organization's recommended minimum 75% tax share of the retail price of tobacco (54, 55). It is important to note one limitation to excise taxes on unhealthy products, even though the primary intent is to curb unhealthy behavior, earmarking is unlikely to bring a sustained net increase in revenue due to offsetting, behavioral shifts, or economic adjustments (54). Nonetheless, these excise taxes may provide an overall source of funding during fiscal constraints and help manage the disease burden. It's important to note that as the bill progresses, tax rates could be adjusted before its final issuance, which may influence the validity of our current analysis. Considering balanced evidence on the advantages, drawbacks, and potential impacts of adopting earmarked excise taxes to fund UHC will be presented in the following section.

#### 3.2.2 Step 2: assembling evidence

In search for evidence behind UHC and financing strategies, a comprehensive document analysis was developed and relied on the highest form of evidence using systematic reviews, scoping reviews, and other peer-reviewed articles. These sources examined the impact and benefits of excise tax adoption earmarked to fund UHC, legal frameworks, and revenue generation for financing UHC and similar health activities. Additional documents from the MoPH, other relevant ministries, universities, public databases, and reports from international institutions further supplement these findings. This approach aligns with Bardach & Patashnik's method of gathering valuable data for policy analysis (56).

##### 3.2.2.1 Evidence of generating revenues through excise taxes to fund health activities and UHC

Earmarking allocates all or a portion of tax revenues for a specific use, such as funding healthcare initiatives. It is gaining momentum as a tool for many health-related goals such as financing the health sector, supporting other health priorities, and funding progress toward UHC (57). Evidence from successful country initiatives in leveraging excise taxes to generate income for health-related activities and UHC has been proven (58). For instance, Partos et al. (59) explained that the three types of government tobacco taxation in the United Kingdom (UK) (ad valorem duty, Value Added Tax, specific excise duty) raise substantial income for the government, with a portion earmarked for funding the NHS. A comparative study on sin tax policies in selected countries for sustainable health financing revealed that Australia increased its tobacco tax from 25% to 30% of the wholesale price, with the resulting revenues directed toward promoting public health initiatives (60).

In LMICs, South Africa has designated specific funds from excise taxes to enable the implementation of a national plan for the Human Immunodeficiency Virus/ Acquired Immunodeficiency Syndrome outbreak (57). Egypt, another African country, earmarked a tax on tobacco covering around 1% of the government expenditure in 2013 and was used to support preventive health programs, particularly for students (61). Additionally, in Vietnam, the Tobacco Control Fund uses a dedicated 1% of tobacco tax income to finance health promotion activities (57).

The above examples demonstrate the effectiveness of excise taxes in generating revenues earmarked for health-related activities like health promotion, with several countries adopting earmarking to dedicate revenue specifically to achieving UHC. For instance, in the Philippines a sin tax reform law was issued in 2012 and focuses on the taxation of tobacco products and alcohol, aiming to fund UHC (62). As well, in Turkey, the Health Transformation Program was financed via a tax-based fund that augmented financial resources and contributed to the achievement of UHC (63).

A debate however exists around the use of earmarked taxes, including earmarking health taxes for specific programs such as UHC (54, 57). Critics argue that earmarking can create rigidity in budgeting, leading to inefficiency and reduced funding for possibly higher-priority or emerging needs. In the case of health financing, earmarking can also increase budgetary fragmentation by creating separate revenue streams for health, which may undermine pooling mechanisms and separate health policies from other sectors that are equally important for improving public health (57). Additionally, some argue that earmarked health taxes are regressive, placing a disproportionate burden on low-income groups through flat excise taxes (57, 64).

Yet, a growing body of evidence in favor of earmarked health taxes challenges these concerns and highlights potential benefits when carefully designed (54, 57, 65). In the context of fiscal constraints, soft earmarking, which tie revenues to priorities less rigidly and allow for some reallocation, are preferred over hard earmarking (54). This approach allows governments greater adaptability to respond to fiscal needs and priorities, while also enhancing accountability, improving allocative efficiency, and fostering public and political support for health taxes (57). Meaning, earmarking guarantees funding for a stated government priority and shields specific revenues from being diverted by competing political interests. Similarly, earmarking can increase public acceptance of taxation by visibly linking it to a specific program, while also alleviating concerns about its regressive nature. For instance, low-income groups who are arguably more responsive to price increases, tend to reduce their consumption of harmful products more than high-income groups in response to price increases, thereby facing a comparatively lower tax burden while realizing greater health and economic benefits through improved behaviors (57). By reducing consumption of harmful products, such taxes can lower healthcare costs for both individuals and governments, while improving overall population health. This dynamic has been particularly evident in the case of tobacco taxes, where cost savings and health gains were found to favor low-income groups compared to their higher-income counterparts (65). As such, linking taxation to a certain program might make people feel more connected to the tax system and reduce resistance (57).

Overall, earmarked health taxes offer significant potential when strategically designed to reflect a country's specific needs and context, effectively aligning sustainable revenue generation and equity goals with the advancement of UHC.

##### 3.2.2.2 Evidence for the establishment of a legal framework for excise taxes

Governments view taxes as effective tools for controlling the consumption of unhealthy products and generating fiscal revenue (63, 66). Systematic reviews and WHO evidence indicate that implementing these taxes as part of system-level policies, coupled with firm government commitment and support, can enhance their effectiveness in securing incomes (66, 67). Evidence from several countries illustrates the advantages of establishing a legal framework for excise taxes used to fund UHC as documented in the literature (58).

A scoping review emphasized that using financial measures, like excise taxes, is necessary to address NCDs such as cardiovascular diseases and diabetes (68). In the United Kingdom (UK), policy implementation via legislation, considered as upstream intervention, has been successful in reducing salt intake among the population as indicated by Hyseni et al. (69). From the context of LMICs, a scoping review that aimed to identify, map and highlight potential gaps in LMIC policy processes related to fiscal measures found that using financial measures, like excise taxes, is necessary to handle NCDs such as cardiovascular diseases and diabetes (68). A study conducted in Sub-Saharan Africa stressed the importance of instituting legal support for these taxes or interventions to ensure they are effective and properly implemented (70). It also focused on the importance of reviewing existing laws before introducing a tax on sugary drinks, which would allow additional money collection to be earmarked for health purposes (70). Moreover, the enactment of tax reforms in the Philippines directly led to a decrease in tobacco use (57).

#### 3.2.3 Step 3: constructing alternatives

The country's current situation presents opportunities to secure funding for UHC. Although existing laws impose taxes on tobacco and alcohol, the absence of an adequate legal framework for earmarking limits effective resource allocation for healthcare. The revised version of the UHC bill exists and the national health strategy recognizes raising funds via earmarked excise taxes, however details about tax rates and revenue allocation remain unclear.

This section presents three potential policy alternatives for allocating excise tax revenue toward UHC in Lebanon. These alternatives are derived from a structured analysis that incorporates evidence from international experiences previously discussed in this paper, as well as insights from prior policy dialogues and briefs conducted by the K2P Center (49, 58, 71). By synthesizing insights from these sources, this section provides feasible and contextually relevant policy options that will be further analyzed.

##### 3.2.3.1 Option 1: establishing an independent UHC fund

This option involves creating a separate, autonomous fund specifically for UHC. The fund would be governed by a board of trustees and have the responsibility to oversee the collection of taxes designated for healthcare and manage the distribution of these revenues directly to UHC programs. This option involves creating an entirely separate, standalone fund exclusively for UHC, managed by a board and subject to its own rules for revenue allocation and accountability.

##### 3.2.3.2 Option 2: legislative framework for earmarking excise tax revenues towards UHC

This alternative focuses on enacting legislation that mandates all excise tax revenues specified in the bill to be allocated directly to UHC only. It would ensure that any funds generated from excise taxes mentioned in the bill are automatically and exclusively channeled toward supporting UHC programs. Unlike Option 1, there would be no separate fund or investment strategy, but the legislative guarantee would ensure that revenues from excise taxes are earmarked solely for the UHC program. This can serve as a quick and immediate step as part of the legislative framework.

##### 3.2.3.3 Option 3: integration of UHC funding into the MoPH budget

This option would integrate excise tax revenue directly into the MoPH's budget. Rather than creating a separate fund or requiring specific legislation for earmarking funds, excise tax revenues would flow into the MoPH's general budget and be used for all health-related expenditures within the ministry, including UHC. This approach offers administrative simplicity, as the MoPH would manage all healthcare financing, but there may be less transparency or protection to ensure that the funds are dedicated exclusively to UHC initiatives.

#### 3.2.4 Step 4 and 5: selecting criteria and projecting outcomes

Steps 4 is used to identify the criteria needed to evaluate the outcomes of policy alternatives, not just assessing the alternatives themselves (26). These criteria help measure how well each alternative performs once adopted and implemented, accounting for the real-world effects of the policy. Step 5 involves projecting the outcomes that are of interest to us. By combining both steps, metrics (qualitative or quantitative) are effectively used to measure and project the success of each alternative, providing clear standards for evaluating whether each policy meets its goals (26). The criteria and outcomes of the three alternatives are presented below:

##### 3.2.4.1 Option 1: establishing an independent UHC fund

**Outcome 1:** The fund ensures that excise tax revenues are channeled directly towards UHC programs.

**Evaluation Criteria**: Is the UHC program well-financed and able to expand coverage and improve healthcare quality as a result of the fund?

**Indicator 1:** Percentage of total excise tax revenue allocated to UHC programs.

**Indicator 2:** Number of actuarial reports assessing UHC fund sustainability.

**Outcome 2:** Weak accountability and transparency in the collection and allocation of funds towards UHC without a legal framework.

**Evaluation Criteria:** Is the governance structure effective in ensuring transparency in fund allocation?

**Indicator 1:** Frequency and enforcement of financial audits.

**Indicator 2:** Publicly available reports on fund allocation and spending.

**Outcome 3:** Delayed establishment and operation of the independent fund.

**Evaluation Criteria:** How costly and complex is it to establish and manage the independent fund?

**Indicator 1:** Number of political endorsements or approvals needed for the creation of the fund.

**Indicator 2:** Public support for the independent UHC fund (measured through polls).

##### 3.2.4.2 Option 2: legislative framework for earmarking excise Tax revenues towards UHC

**Outcome 1:** Direct allocation of excise taxes toward UHC.

**Evaluation Criteria:** Does the legislation ensure that all collected excise tax revenues are effectively directed toward UHC in a timely manner?

**Indicator 1:** Percentage of excise tax revenue allocated to UHC programs.

**Indicator 2:** Total revenue generated annually by health-related taxes.

**Indicator 3:** Time taken for fund allocation to UHC programs from point of revenue collection to disbursement

**Outcome 2:** Improvement in healthcare coverage and access.

**Evaluation Criteria:** Does the direct allocation of excise taxes result in expanded healthcare coverage?

**Indicator 1:** Increase in the percentage of the population covered by UHC.

**Indicator 2:** Number of additional healthcare facilities or services provided due to UHC funding.

**Indicator 3:** Reduction in out-of-pocket expenses for healthcare among citizens.

**Outcome 3:** The legal framework provides transparency in the allocation of funds.

**Evaluation Criteria:** Is there clear reporting and tracking of how funds are used for UHC?

**Indicator 1:** Number of public reports on excise tax revenue and UHC expenditures.

**Indicator 2:** Existence of oversight mechanisms (e.g., independent audit bodies) ensuring that excise tax revenues are directed to UHC.

##### 3.2.4.3 Option 3: integration of UHC funding into the MoPH budget

**Outcome 1:** The MoPH has flexibility in allocating resources based on healthcare priorities.

**Evaluation Criteria:** Does the flexibility of the MoPH budget allow it to respond to emerging healthcare needs while adequately supporting UHC?

**Indicator 1:** Percentage of the MoPH budget allocated to UHC initiatives compared to other health programs.

**Indicator 2:** Number of UHC programs expanded or adapted based on emerging healthcare challenges.

**Outcome 2:** UHC funding may be diluted as it is integrated into the broader MoPH budget.

**Evaluation Criteria:** How effectively can the MoPH ensure that UHC programs receive sufficient and dedicated funding?

**Indicator 1:** Percentage of excise tax revenue allocated specifically to UHC within the broader MoPH budget.

**Indicator 2:** Occurrence of budget reallocations away from UHC to other health initiatives.

**Indicator 3:** Stakeholder feedback (e.g., UHC program administrators) on whether UHC funding is sufficient.

**Outcome 3:** Less political resistance due to the use of existing structures.

**Evaluation Criteria:** Is this option politically easier to implement because it uses existing MoPH structures?

**Indicator 1:** Level of public and political support for integrating UHC funds into the MoPH budget.

**Indicator 2:** Number of policy or legislative approvals required to implement this approach.

#### 3.2.5 Step 6: confronting trade-offs

##### 3.2.5.1 Option 1

In the Lebanese context, where political fragmentation and frequent governmental turnover can lead to instability, an independent fund might provide protection from political shifts and ensure a consistent flow of resources dedicated to health, without having to amend the existing constitution. However, the primary concern lies in ensuring transparency and accountability, especially given Lebanon's history of governance challenges, including corruption and a lack of public trust (38, 72). Unlike a government-managed system, where established oversight mechanisms (such as audits by the Ministry of Finance) could be applied, an independent fund may be more vulnerable to mismanagement or lack of effective oversight. Additionally, the development of this independent fund would be costly, time consuming, and would require political will to be established.

##### 3.2.5.2 Option 2

A robust legislation framework ensures that excise taxes flow transparently through the national treasury to the UHC program holding the government accountable for achieving its goals. This approach directly supports UHC without the administrative burden of creating and managing a separate fund. The framework also allows for the rapid expansion of UHC coverage and services, ensuring that a larger percentage of the population benefits quickly from the policy. In terms of transparency, Option 2 provides strong oversight mechanisms, but without the added complexity and cost. Politically, although this option requires the passage of new legislation, it is less complicated than establishing an independent fund and can be implemented more swiftly, minimizing delays.

##### 3.2.5.3 Option 3

The integration of excise tax revenue into the entire MoPH budget simplifies resource management and ensures dedicated excise taxes directly support UHC. However, potential competition with existing health priorities within the MoPH budget is a concern. Additionally, UHC funding may be diluted as it is integrated into the broader MoPH budget. This could reduce the focus on expanding UHC programs and make it difficult to ensure that UHC receives sufficient and dedicated funding. Although this option faces the least political resistance, as it utilizes existing structures, the risk of fund dilution and the lack of clear accountability make it a less reliable choice for achieving comprehensive healthcare coverage.

#### 3.2.6 Step 7: decision-making

Considering the above options, **Option 2** emerges as the most balanced and strategic choice. While it may face initial delays due to the need for regulatory and possibly constitutional reforms, it offers the strongest legal foundation for earmarking excise taxes and ensures transparent oversight through the treasury. This approach aligns with best practices from other countries that have successfully implemented earmarking for excise taxes, making it a solid long-term solution for funding UHC in Lebanon. It stands out as the most effective policy choice, balancing immediate impact on healthcare access, clear accountability, and political feasibility (73). It ensures that all excise tax revenues are legally bound to support UHC programs directly, providing fast and reliable improvements to healthcare coverage and access without the complexity and potential delays of an independent fund (Option 1) or the risks of fund dilution in the broader MoPH budget (Option 3).

## 4 Discussion

This paper used John Kingdon's MSF to understand how the three streams of problem, policy, and politics converged opening a window of opportunity that put the proposed UHC bill at the forefront of the policy agenda in Lebanon. Multiple crises, inflation, and increased healthcare costs characterized the problem stream, while the policy stream involved proposing solutions, such as the National Health Strategy and the Health Transformation Project, both developed through collaborative efforts to address healthcare gaps and advance UHC. The politics stream included political dynamics, will, and change in political leadership, all of which contributed to the prioritization of the UHC bill in Lebanon. Also, it stresses the role of actors and stakeholders and their engagement during research processes for UHC policy and political decision-making.

A policy analysis of the proposed UHC bill in Lebanon was conducted from a legal perspective, focusing on earmarking excise taxes as a potential funding mechanism for UHC. Using Bardach & Patashnik's Policy Analysis Framework, an analysis of trade-offs among three funding alternatives for UHC identified Option 2 (establishing a legislative framework to earmark excise tax revenues for UHC) as the most suitable choice. This analysis is grounded in Lebanon's current constitutional and fiscal legal framework, which directly influences the feasibility of earmarking excise tax revenues for UHC. The Lebanese Constitution, specifically Articles 81–83, governs taxation and public revenue collection and explicitly requires legislation to earmark public revenues (74). Accordingly, enacting dedicated legislation to earmark excise taxes for funding UHC is a requirement following agenda setting (74). Furthermore, the Lebanese Public Accounting Law (Decree No. 14969/1963) provides relevant provisions that, while not explicitly using the term “earmarking”, enable the allocation of revenues to specific expenditures through mechanisms such as special accounts and exceptional appropriations funded by designated resources (e.g., Articles 36, 61, 95, and 102) (75). These articles collectively provide a legal basis for allocating specific revenue such as those from health-related taxes to targeted expenditures like UHC, provided that the necessary legal authorizations are in place. As such, the proposed 2023 UHC Bill is central to this analysis, with Article 7 detailing the proposed excise tax structure and intended allocation mechanisms. This draft law represents an opportunity to operationalize earmarking in line with both constitutional requirements and existing public finance procedures. Together, these documents form the legal foundation of the policy analysis presented in this study and offer a reference point for future comparative work as Lebanon's health financing legislation evolves.

The WHO has previously described civil society movements and advocacy efforts as powerful drivers towards UHC (76). With the UHC bill now on the Lebanese policy agenda, sustained advocacy is essential to move it toward enactment and implementation. Anticipated challenges include resistance from business owners, complex coordination between public and private facilities, and the historical lack of political will in Lebanon. Developing a structured, evidence-based advocacy strategy is therefore crucial, as it fosters political commitment and mobilizes the resources needed for health system reform (77). Frameworks such as the Public Health Advocacy Institute of Western Australia (78) and the UHC 2030 advocacy guide (77) can inform these efforts by combining evidence generation, stakeholder mapping, and targeted strategies to overcome opposition and ensure the successful adoption and implementation of the UHC bill. Examples from European countries include sociopolitical movements such as the strike waves in France, which pushed for the development of social benefits (79). Similarly in Latin America, the reform of the Brazilian health sector was driven by civil societies instead of governments, political parties, or international organizations (79). This is no different than scenarios in the Middle East, where Turkey's history showed sociopolitical changes and paved the way towards equitable healthcare services including the introduction of the Law on the Nationalization of Health Care Delivery (Law Number 224) and the Law on Population Planning (Law Number 554) (79). More recently, the enactment of the new UHC law in Egypt was also driven by advocacy efforts from a diverse set of actors with varying interests and levels of influence (80). Even though the ministry of finance had the right to veto, none state actors such as NGOs, syndicates, and labor unions, were part of the law drafting committee (80). Social engagement and advocacy are thus integral to advancing UHC in Lebanon, serving as both prerequisites and enablers of progress (80).

Less than half of the countries in the Middle East and North Africa (MENA) region have formally enacted legislation supporting UHC, with Tunisia, Oman, and the United Arab Emirates being among them (81). Tunisa, who like Lebanon, has a history of political instability and a deteriorating economic situation, has placed a National Health Policy which intended to achieve UHC by 2030 (82). Similar to Tunisia, Lebanon's path to UHC has been shaped by crises, yet similar challenges can be seen, including fragmented health financing and financial barriers to care. In both countries, the sustainability of UHC efforts is constrained by a small tax base, limited public health funds, and gaps in health coverage and financial protection, particularly for nonpoor informal workers (83).

Despite facing similar challenges, including a fragmented healthcare system, Egypt enacted the Universal Health Insurance (UHI) law in 2019, marking gradual progress toward UHC (84) and increasing the UHC service index from 50 in 2000 to 70 in 2021 (85). A key strength of Egypt's approach has been the establishment of three autonomous organizations: (1) the Universal Health Insurance Authority (UHIA), which pools funds and purchases health services on behalf of the insured population; (2) the Healthcare Organization, responsible for healthcare service provision; and (3) the General Authority for Healthcare Accreditation and Regulation, which ensures financial sustainability (86). Lebanon has been closely working with experts from Egypt and drawing lessons from its health financing mechanisms and institutional frameworks. These learnings have shaped Lebanon's progress toward UHC and will continue to guide reforms until full implementation is achieved.

The WHO has emphasized the importance of UHC in promoting health equity by ensuring financial protection and reducing catastrophic OOP expenditures, an issue that is gaining momentum in Lebanon following the recent introduction of the UHC bill to the policy agenda (1). For effective and sustainable health financing reform, governments in the Arab world should apply an analytical framework centered on four essential elements: revenue generation (e.g., who contributes and when), pooling (who receives the benefits), purchasing (which services are paid for by beneficiaries), and benefit package design (what services are covered) (73). In response to Lebanon's crises, a recommended evidence-based approach to increase government revenues for financing UHC is the implementation of earmarked excise taxes, supported by a legislative framework that ensures all excise tax revenues specified in the bill are allocated directly to UHC.

The strengths of this study include utilizing two well-regarded theoretical frameworks: John Kingdon's MSF and Bardach & Patashnik's Policy Analysis Framework. This allows for a comprehensive examination of both the agenda-setting process and the funding mechanisms for the UHC bill, ensuring the study is grounded in widely accepted policy analysis methodologies. The analysis of excise taxes and their role in funding UHC provides valuable insights into the fiscal challenges and opportunities for sustainable healthcare financing in Lebanon. This legal perspective, combined with international examples of earmarking excise taxes, adds depth to the analysis and strengthens the policy recommendations. Coupled with a comprehensive content analysis methodology including systematic reviews, government publications, and international reports, this strengthens the credibility of the findings and ensures a thorough analysis of the policy issues.

However, empirical evidence from qualitative interviews or quantitative surveys with key stakeholders was missing. This study relied on the best available evidence from secondary sources as explained in the methodology section, however primary data collection was neither feasible nor practical given the circumstances in Lebanon. Lebanon's political and economic instability created significant barriers to conducting surveys or interviews, making data collection unreliable. Additionally, the rapidly evolving situation required timely analysis, and the extensive time required for primary data collection would have hindered the study's relevance and applicability. Given the unique political and economic circumstances in Lebanon, the findings and policy recommendations may not be generalizable to other countries. We encourage future studies to broaden the scope beyond excise taxes by exploring other potential funding sources for UHC, such as public-private partnerships, external donor aid, or innovative financing models to provide a more comprehensive set of policy options. It is also important to note that some of the decisions regarding the exact earmarking of excise taxes are not final, and may be subject to changes after the publication of this manuscript. Finally, while earmarking health taxes can secure dedicated funding, enhance accountability, and strengthen support for UHC, it also carries risks such as budget rigidity, reduced flexibility, and potential inequities if poorly designed. Therefore, any earmarking of public revenues should be applied cautiously and framed within a broader, well-governed public financial management strategy that maintains the integrity of the unified budget and allows for annual reassessment of priorities to ensure equitable and sustainable health financing.

## Data Availability

The original contributions presented in the study are included in the article/Supplementary Material, further inquiries can be directed to the corresponding author.
